# Risk assessment of mercury and lead in fish species from Iranian international wetlands

**DOI:** 10.1016/j.mex.2018.05.002

**Published:** 2018-05-05

**Authors:** Ghasem Zolfaghari

**Affiliations:** Department of Environmental Sciences and Engineering, Faculty of Environmental Sciences, Hakim Sabzevari University, Razavi Khorasan, Sabzevar, P.O. Box: 397, Iran

**Keywords:** Risk assessment, Heavy metals, Anzali wetland, Caspian Sea, Hamun wetlands

## Abstract

The aim of this study is determination of mercury concentration in the muscle, intestine, gonad and kidney of *Rutilus rutilus*, *Hemiculter Leucisculus* (Anzali wetland), and *Alosa Caspia Caspia* (Caspian Sea), and mercury and lead concentrations in the muscle of *Ctenopharyngodon idella*, *Cyprinus carpio*, *Hypophthalmichthys molitrix*, *Hypophthalmichthys nobilis*, *Schizocypris altidorsalis*, and *Schizothorax zardunyi* (Hamun wetlands). The results of this study were compared with global standards. As well as in this multispecies monitoring, health risk assessment of consumers by EPA/WHO instructions has been done. The concentrations of mercury were below the limits for fish proposed by United Nations Food and Agriculture Organization (FAO), World Health Organization (WHO), US Food and Drug Administration (FDA) and US Environmental Protection Agency (EPA), and European Union (EU). Lead concentrations in *Ctenopharyngodon idella*, *Cyprinus carpio*, *Hypophthalmichthys molitrix* was under the scope proposed by FAO, WHO, FDA, Turkish Acceptable Limits (TAL), United Kingdom Ministry of Agriculture Fisheries and Food (UK MAFF) and National Health and Medical Research Council (NHMRS), but lead concentration in *Schizocypris altidorsalis*, and *Schizothorax zardunyi* were higher than WHO and TAL. Health risk assessment of consumers from the intake of metal contaminated (mercury and lead) was evaluated by using Hazard Quotient (HQ) calculations. The human health hazard Quotient (index) showed that the cumulative risk greatly increases with increasing fish consumption rate, thus yielding an alarming concern for the consumer’s health.

• The results of the present study aimed to provide data from Caspian Sea, Anzali wetland, and Hamoon wetland as indicators of natural and anthropogenic impacts on aquatic ecosystem as well as to evaluate the human hazard index associated with fish consumption.
• The results show that for mercury, the Maximum Allowable Fish Consumption Rate (Meals/Month) is related to *Hemiculter Leucisculus*.
• The results for lead concentration indicate that there is no HQ value > 1, indicating that humans would not experience any significant health risk if they only consume metals from these species of fish from the hamun wetland.

## Graphical abstract

**Figure:**
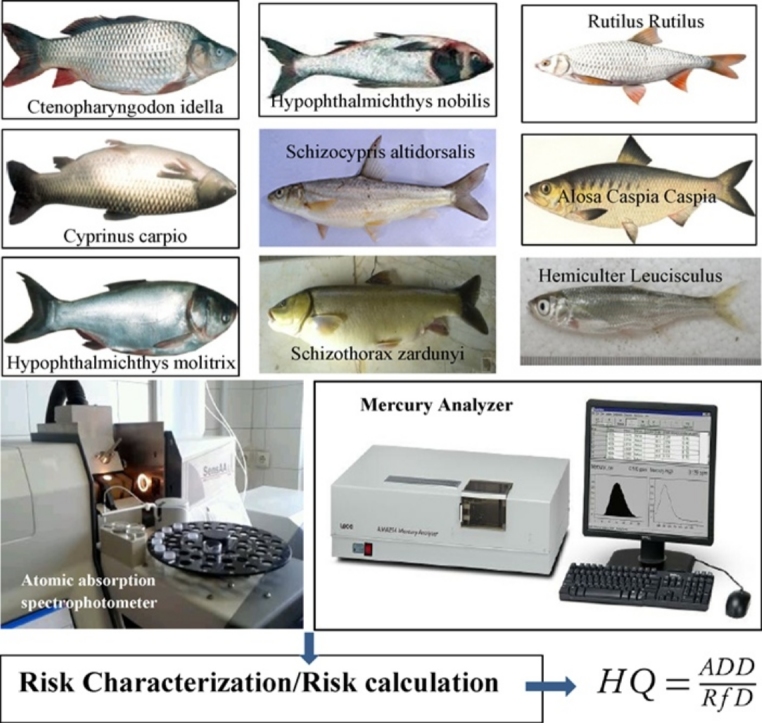

## Methods details

### Case study

**Fig. 1:**
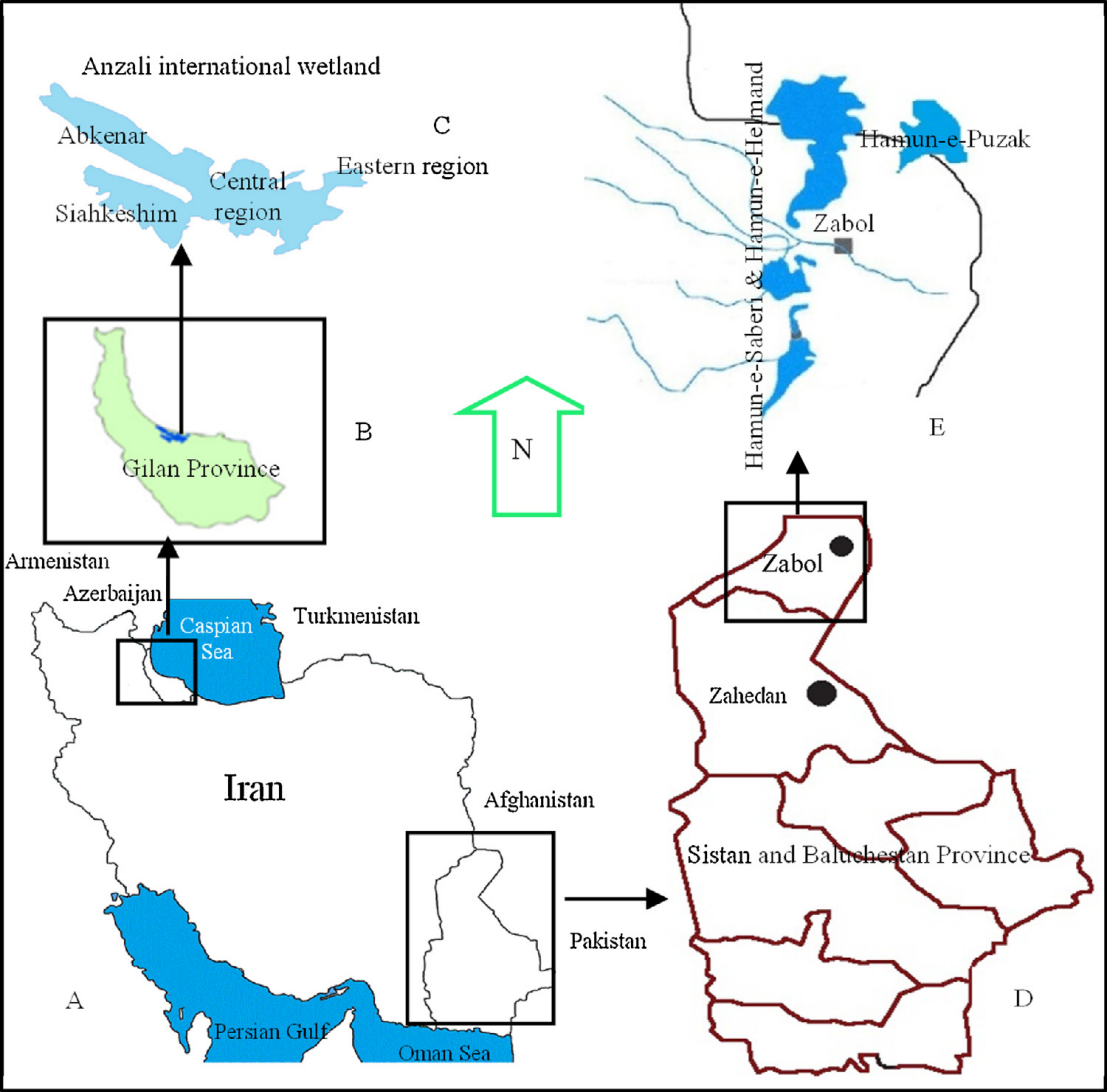
Iran situation (A), Gilan Province (B), Anzali international wetland (C), Sistan and Baluchestan Province (D), Hamun wetlands (E).

The Caspian Sea (Fig. 1), which is located in the northern I.R. Iran, is the largest lake in the world and is connected to the distant Baltic through canals and the River Volga. It is unique closed water basin, plays the important role in the establishment of the climate. The Anzali Wetland (193 km^2^) (Fig. 1), located on the southern coast of the Caspian Sea, is internationally known as an important wetland for migratory birds, and was registered as a Ramsar site in June 1975 in accordance with the Ramsar Convention. Hamun wetland, the largest freshwater expanse of the Iranian plateau, is listed in the Convention on Wetlands, Ramsar [1].

### Sampling

The fish species including Rutilus rutilus, Hemiculter Leucisculus (from Anzali wetland), and *Alosa Caspia Caspia* (from Caspian Sea), Ctenopharyngodon idella, Cyprinus carpio, Hypophthalmichthys molitrix, Hypophthalmichthys nobilis, Schizocypris altidorsalis, and *Schizothorax zardunyi* (Hamun wetland) were randomly collected. Twenty (20) fish samples from each species were transferred to the laboratory and stored in refrigerator. Afterwards, the tissues were separated and dried.

### Mercury and lead analysis

The dried samples were ground and changed into a homogenous powder and then the mercury concentration rate has been determined by Advanced Mercury Analyzer (AMA), LECO AMA 254 according to ASTM, standard No. D-6722. Each sample was analyzed 3 times. The LECO AMA 254 is a unique Atomic Absorption Spectrometer (AAS) that is specifically designed to determine total mercury content in various solids and certain liquids without sample pre-treatment or sample pre-concentration. Designed with a front-end combustion tube that is ideal for the decomposition of matrices, the instrument’s operation may be separated into three phases during any given analysis: Decomposition, Collection, and Detection [2].

**Fig. 2:**
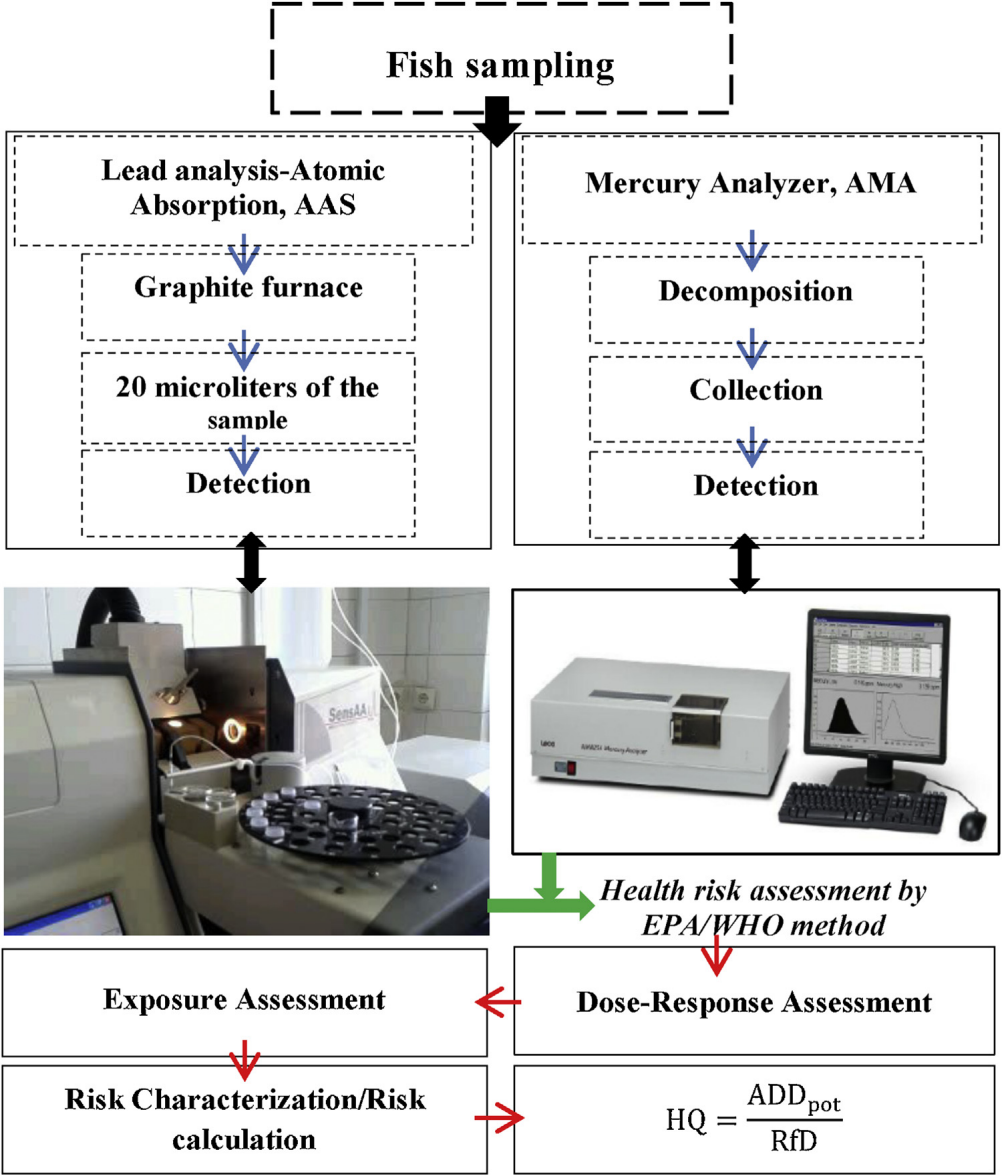
Diagram presenting the steps of the procedures used in this study.

The AAS equipped with graphite furnace (GBC GF 3000 model) was used for lead analysis. A volume of 20 microliters of the sample was injected into the device [3]. Fig. 2 shows the steps of the procedures used in this study.

### Quality control

In order to assess the analytical capability of the AMA methodology, accuracy of total mercury analysis was checked by running three samples of Standard Reference Material (SRM), NIST (National Institute of Standards and Technology) SRM 1633b, SRM 2709, and SRM 2711 in six replicates. Recovery varied between 95% and 100%. In order to check the reproducibility of the analysis, the samples were analyzed in triplicate. The coefficient of variation was between 0.05% and 2.5%. The accuracy of the AAS method was verified by analyzing the standard reference material 1515-Apple Leaves (NIST). Certified value, observed value, and recovery was 0.470 ± 0.024, 0.450 ± 0.042, and 95.7%, respectively. As it can be seen, there is a good agreement between observed mean and certiﬁed value [4].

### Health risk assessment by EPA/WHO method

There are four steps in this method [5,6]:

#### Hazard identification

Hazard identification involves gathering and evaluating toxicity data on the types of health injury or disease that may be produced by a chemical and the conditions of exposure under which injury or disease is produced. The subset of chemicals selected for the study is termed “chemicals of potential concern”. Data from acute, subchronic, and chronic dose-response studies are used [7].

#### Dose-response assessment

The dose-response assessment involves describing the quantitative relationship between the amount of exposure to a chemical and the extent of toxic injury or disease. The US EPA established the Reference Dose (RfD) as Eq. (1) [5]:

(1) $$RfD=\frac{NOAEL.or.LOAEL}{UF.\times MF}$$

NOAEL: No Observed Adverse Effect Level

LOAEL: Low Observed Adverse Effect Level

UF: Uncertainly Factor

MF: Modifying Factor

#### Exposure assessment

Applies a generalized dose-response relationship to specific conditions for some population. Characterizes the sources of an environmental hazard, concentration levels at that point, pathways, and any sensitivities. Exposure assessment involves describing the nature and size of various populations exposed to a chemical agent, and the magnitude and duration of their exposures. The exposure pathway of heavy metals to human through ingestion of contaminated food has been studied by many researchers [8,9]. Average Daily Dose for Intake Process ${ADD}_{pot}$ is calculated as Eq. (2) [6]:

(2) $${ADD}_{pot}=\frac{.(C\times .IR\times ED)}{BW\times AT}$$

C: Concentration of toxic material

IR: Ingestion Rate

ED: Exposure Duration

BW: Body Weight

AT: Averaging Time

Average Daily Dose for Uptake Process (ADD_int_) is calculated as Eq. (3) [5]:

(3) $${ADD}_{int}=\frac{.(C\times .IR\times ED\times AF.)}{BW\times AT}$$

AF: a fraction of the dose in the organ or tissue that is absorbed after a while. AF for this study was assumed 0.4.

#### Risk characterization/risk calculation

The Average Daily Dose for Intake Process (${ADD}_{pot}$) (Total Intake) is compared to the RfD. If ${ADD}_{pot}$ < RfD, then no problem. Hazard Quotient (HQ) is calculated as Eq. (4) [10]:

(4) $$HQ=\frac{{ADD}_{pot}}{RfD}$$

## Results and discussion

**Fig. 3:**
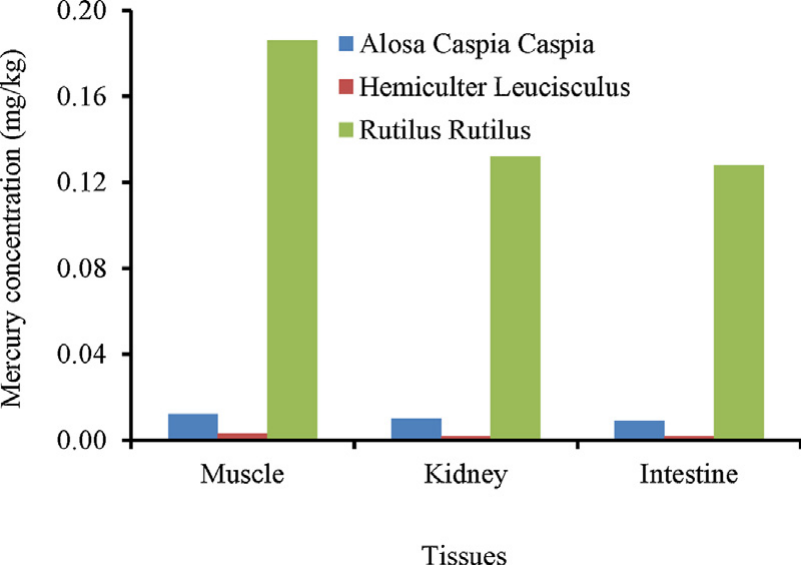
Comparison of mercury concentrations in fishes tissues from Anzali wetland and Caspian Sea.

The concentrations of Hg in tissues of *Rutilus rutilus, Hemiculter Leucisculus*, and *Alosa Caspia Caspia* was measured (Table 1). The results of laboratory analysis showed that there are significant difference between the concentration of mercury between species (*p *< 0.001) (Fig. 3). There was no significant difference between the independent variables of gender, age and weight of the dependent variable is the amount of mercury in the tissues of the *Rutilus rutilus*. But between the length and the amount of mercury in the kidney of *Rutilus rutilus*, there was significant difference at 95% (*p* = 0.015).

**Table 1:** The results of measuring the total mercury concentration in mg/kg (dry weight) in the fish tissues of Anzali Wetland.

Species name		Tissues	Mercury concentration (mg/kg)	Minimum	Maximum
English name	Scientific name				
North caspian roach	*Rutilus Rutilus*	Muscle	0.18 ± 0.043	0.087	0.26
		Kidney	0.13 ± 0.064	0.076	0.37
		Gonad	0.13 ± 0.028	0.097	0.28
		Intestine	0.12 ± 0.046	0.077	0.18
Caspian shad	*Alosa Caspia Caspia*	Muscle	0.012 ± 0.005	0.006	0.017
		Kidney	0.010 ± 0.002	0.005	0.015
		Intestine	0.009 ± 0.002	0.004	0.013
Sharpbelly	*Hemiculter Leucisculus*	Muscle	0.003 ± 0.0005	0.001	0.009
		Kidney	0.002 ± 0.0005	0.001	0.007
		Intestine	0.002 ± 0.0005	0.001	0.006

**Fig. 4:**
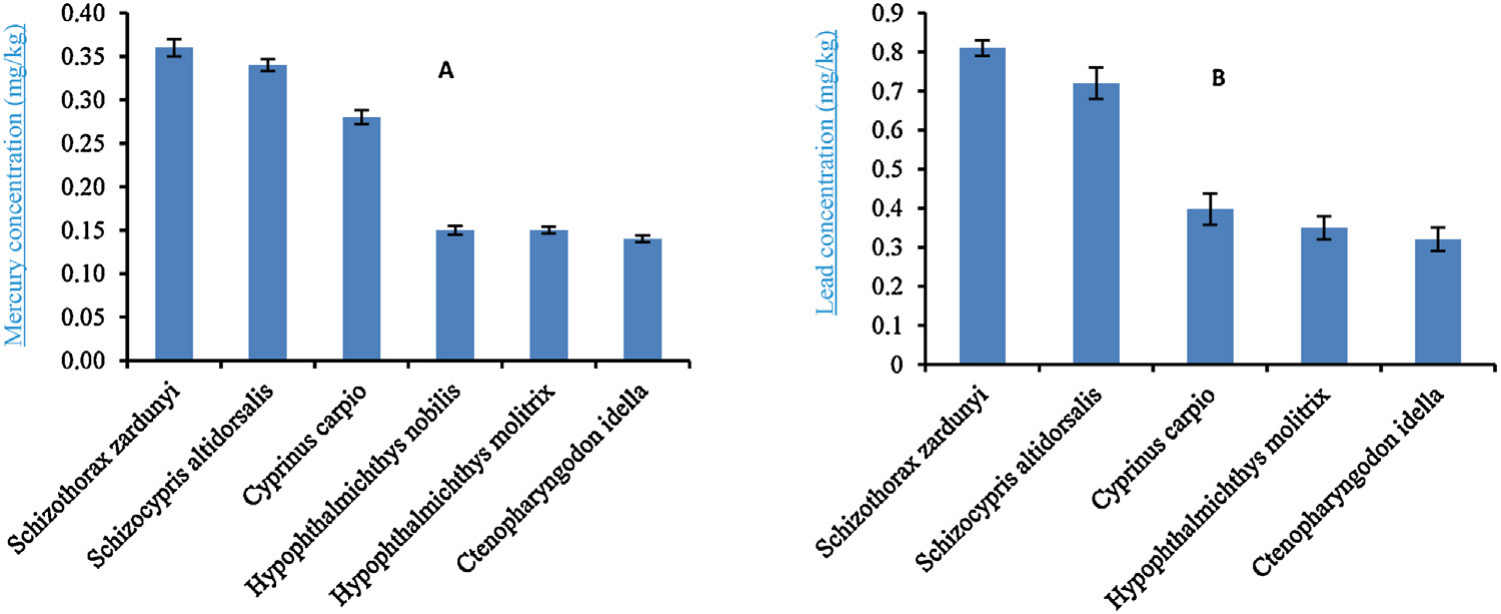
Comparison of mercury concentrations (figure A) and lead (right figure B) in fishes muscle from Hamun wetlands.

Mean concentrations of Hg in muscle of Ctenopharyngodon idella, Cyprinus carpio, Hypophthalmichthys molitrix, Hypophthalmichthys nobilis, Schizocypris altidorsalis*, and Schizothorax zardunyi* were 0.14, 0.28, 0.15, 0.15, 0.34 and 0.36 mg/kg respectively (Table 2). The results of laboratory analysis showed that there are significant difference between the concentration of mercury in the muscle between species (*p < *0.001) (Fig. 4A). Mean concentrations of Pb in muscle of Ctenopharyngodon idella*, Cyprinus carpio*, Hypophthalmichthys molitrix, Schizocypris altidorsalis, and *Schizothorax zardunyi* were 0.32, 0.39, 0.35, 0.72 and 0.81 mg/kg respectively (Table 2). There was no significant difference between lead concentrations of these species (*p > *0.05) (Fig. 4B).

**Table 2:** The results of measuring the mercury and lead concentrations in mg/kg (dry weight) in the fish tissues of Hamun Wetland.

English name	Scientific name	Mercury concentration (Muscle)			Lead concentration (Muscle)		
		Mean	Minimum	Maximum	Mean	Minimum	Maximum
Grass carp	*Ctenopharyngodon idella*	0.14 ± 0.004	0.12	0.19	0.32 ± 0.03	0.13	0.65
Common crap	*Cyprinus carpio*	0.28 ± 0.008	0.23	0.35	0.39 ± 0.04	0.17	0.76
Silver carp	*Hypophthalmichthys molitrix*	0.15 ± 0.004	0.12	0.20	0.35 ± 0.03	0.10	0.85
Bighead	*Hypophthalmichthys nobilis*	0.15 ± 0.005	0.10	0.20	–	–	–
–	*Schizocypris altidorsalis*	0.34 ± 0.007	0.29	0.40	0.72 ± 0.04	0.30	0.99
–	*Schizothorax zardunyi*	0.36 ± 0.01	0.23	0.46	0.81 ± 0.02	0.60	0.98

Table 3 shows ADD_pot_ and HQ of heavy metals in muscles of fish samples from the wetlands. Among the fish species examined in this study, *Hemiculter Leucisculus* with a HQ value of 0.009 has the lowest potential health risk to mercury and *Schizothorax zardunyi* with a HQ value of 1.2 has the highest potential health risk to mercury.

**Table 3:** Average Daily Dose for Intake Process (${ADD}_{pot}$), Average Daily Dose for Uptake Process (ADD_int_), Hazard Quotient (HQ), and Maximum Allowable Fish Consumption Rate Limit (CR_lim_).

Fish species	Mercury**					Lead					
	Mercury concentration of muscle (mg/kg)	${ADD}_{pot}$ (μg/kg/day)	HQ	CR_lim_ (kg/d)	CR_mm_*** Meals/Mo	Lead concentration of muscle (mg/kg)	${ADD}_{pot}$ (μg/kg/day)	ADD_in_ (μg/kg/day)	HQ	CR_lim_ (kg/d)	CR_mm_*** Meals/Mo
*Rutilus Rutilus*	0.18	0.06	0.6	0.038	5.09	Not measured	–	–	–	–	–
*Alosa Caspia Caspia*	0.012	0.04	0.4	0.583	17.74	Not measured	–	–	–	–	–
*Hemiculter Leucisculus*	0.003	0.0009	0.009	2.333	312.84	Not measured	–	–	–	–	–
*Ctenopharyngodon idella*	0.14	0.05	0.5	0.050	6.70	0.32	0.10	0.05	0.0003	30.62	4106
*Cyprinus carpio*	0.28	0.09	0.9	0.025	3.35	0.39	0.13	0.07	0.0005	12.25	1642
*Hypophthalmichthys molitrix*	0.15	0.05	0.5	0.046	6.16	0.35	0.11	0.06	0.0004	28.00	3754
*Hypophthalmichthys nobilis*	0.15	0.05	0.5	0.046	6.16	Not measured	–	–	–	–	–
*Schizocypris altidorsalis*	0.34	0.11	1.1	0.020	2.68	0.72	0.23	0.12	0.0008	42.60	5712
*Schizothorax zardunyi*	0.36	0.12	1.2	0.019	2.54	0.81	0.26	0.13	0.0009	12.09	1621
Mean (Anzali fishes)	0.06	0.02	0.2	0.116	15.55	–	–	–	–	–	–
Mean (Hamun fishes)	0.23	0.08	0.8	0.030	4.02	0.51	0.16	0.09	0.0006	19.21	2576

^*^ The amount of fish consumption is 29.23 g / day, according to the statistics of the Iranian Fisheries Organization.

** For mercury, ADD_op_ is considered to be ADD_int._.

*** The amount of fish consumed per meal is 0.227 kg, according to the US EPA Guide.

The HQ through the consumption of *Schizocypris altidorsalis* and *Schizothorax zardunyi* was higher than 1 (for mercury), indicating that there is potential health risk associated with the consumption of these fish from the hamun wetland. The results for lead concentration indicate that there is no HQ value>1, indicating that humans would not experience any significant health risk if they only consume metals from these species of fish from the hamun wetland.

The concentrations of mercury in all species were below the limits for fish proposed by United Nations Food and Agriculture Organization (FAO), World Health Organization (WHO), US Food and Drug Administration (FDA) and US Environmental Protection Agency (EPA), and European Union (EU) (Table 4). Lead concentrations in *Ctenopharyngodon idella, Cyprinus carpio, Hypophthalmichthys molitrix* were under the scope proposed by FAO, WHO, FDA, Turkish Acceptable Limits (TAL), United Kingdom Ministry of Agriculture Fisheries and Food (UK MAFF) and National Health and Medical Research Council (NHMRS), but lead concentration in *Schizocypris altidorsalis, and Schizothorax zardunyi* were higher than WHO and TAL (Table 5).

**Table 4:** Threshold Levels of mercury and lead in fish muscle tissue (mg/kg).

Standard	Lead	Reference	Standard	Reference	Mercury
World Health Organization (WHO)	0.5	[11]	World Health Organization (WHO)	[17]	0.5
Food and Agriculture Organization (FAO)	2	[12]	Food and Agriculture Organization (FAO)	[18]	2
Ministry of Agriculture, Fisheries and Food (MAFF)	2	[13]	United States Environmental Protection Agency (US EPA)	[19]	2
National Health and Medical Research Council (NHMRS)- United Kingdom	1.5	[14]	Europe Commission (EC)	[20]	1.5
United States Food and Drug Administration (US FDA)- Australia	5	[15]	China	[12]	5
Turkish Acceptable Limits (TAL)	0.4	[16]	United States Food and Drug Administration (US FDA)	[21]	0.4

**Table 5:** Values set by reference agencies for the concentration of mercury and lead (μg/kg/day).

PTDI (WHO) Mercury	PTWI (WHO)) Mercury	PTDI (US FDA)) Mercury	PTWI (US FDA)) Mercury	PTDI (US EPA) Mercury
0.0007	0.0049	0.0004	0.0028	0.0001
PTWI (US EPA) Mercury	RfD Mercury	LOAEL Mercury	PTDI (WHO) Lead	RfD Lead
0.0007	0.1	3	3.07	140

The daily allowable consumption rate of fish is calculated according to the amount of pollutant stored in the oral area (muscle) by the Eq. (5) proposed by the US EPA [19]:

(5) $$\text{C}{\text{R}}_{\lim}=\frac{\text{RfD}\times \text{BW}}{C_{\text{m}}}$$

CR_lim_ (kg/day) is Maximum Allowable Consumption Rate per day (Table 3). The highest amount of allowable consumption regarding mercury is for *Hemiculter Leucisculus* (2.33 kg/day). In contrast, *Schizothorax zardunyi* has the lowest amount of fish intake (0.019 kg/day). It should be noted that maximum consumption of 0.020 kg/day of *Schizocypris altidorsalis* and 0.019 kg/day of *Schizothorax zardunyi* there is no potential health risk (CR_lim_).

CR_lim_ can also be used to determine the Maximum Allowable Fish Consumption Rate per month (Meals/Month (CR_mm_)) using Eq. (6) [19]:

(6) $$\text{C}{\text{R}}_{\text{mm}}=\frac{\text{C}{\text{R}}_{\text{lim}}\times {\text{T}}_{\text{ap}}}{\text{MS}}$$

T_ap_: time averaging period (365 days per year and 30.44 days per month)

MS: meal size (0.227 kg for adults)

The results show that for mercury, the Maximum Allowable Fish Consumption Rate (Meals/Month) is related to *Hemiculter Leucisculus*.

## Conclusion

The results of the present study aimed to provide data from Caspian Sea, Anzali wetland, and Hamoon wetland as indicators of natural and anthropogenic impacts on aquatic ecosystem as well as to evaluate the human hazard index associated with fish consumption. The results indicated that the highest Average Daily Dose regarding mercury was for *Schizocypris altidorsalis* and *Schizothorax zardunyi*. The Maximum Allowable Fish Consumption Rate per month for mentioned fishes was 2.68 and 2.54 meals, respectively. This result regarding lead for *Schizocypris altidorsalis* was interesting (5712 meals/month). The human health Hazard Quotient showed that the cumulative risk greatly increases with increasing fish consumption rate, thus yielding an alarming concern for consumer health. The annual monitoring and measurement of heavy metals and other pollutants in fishes of wetlands and production of a database is necessary.

## Additional information

In between aquatic ecosystems, wetlands and rivers have a great ecological importance. Heavy metals from geological and anthropogenic sources are increasingly being released into natural waters. Contamination of aquatic ecosystems with heavy metals has seriously increased worldwide attention, and a lot of studies have been published on the heavy metals in the aquatic environment. Under certain environmental conditions, heavy metals may accumulate to toxic concentrations and cause ecological damage [22]. Mercury is a special concern in marine ecosystems, where methylation occurs during the process of biotransformation and accumulates in biota. Mercury is a toxin to the central nervous system and it can readily cross the placental barrier [23]. Lead is attracting wide attention of environmentalists as one of the most toxic heavy metals. The sources of lead release into the environment by waste streams are battery manufacturing, acid metal plating and finishing, ammunition, tetraethyl lead manufacturing, ceramic and glass industries printing, painting, dying, and other industries. Lead has been well recognized for its negative effect on the environment where it accumulates readily in living systems. Lead poisoning in human causes severe damage to the kidney, nervous system, reproductive system, liver and brain [24]. The results of a study in Khur-e-Khuran international wetland in the Persian Gulf, Iran show that measured values of most heavy metals in some examined fishes of Khur-e-Khuran wetland were higher than those maximum permissible limit according to international standards [25].

The aim of this study is determination of mercury concentration in the muscle, intestine, gonad and kidney of *Rutilus rutilus, Hemiculter Leucisculus* (Anzali wetland), and *Alosa Caspia Caspia* (Caspian Sea), and mercury and lead concentrations in the muscle of *Ctenopharyngodon idella, Cyprinus carpio, Hypophthalmichthys molitrix, Hypophthalmichthys nobilis, Schizocypris altidorsalis,* and *Schizothorax zardunyi* (Hamun wetland). The results of this study were compared with global standards. In this multispecies monitoring, health risk assessment of consumers by EPA/WHO instructions has been done. The main objective was to evaluate the potential health risks associated with heavy metals via consumption of fish from the wetlands using the Average Daily Dose for Intake Process (${ADD}_{pot}$) and Hazard Quotient (HQ) from heavy metals. This paper provides the first quantitative information on accumulation of mercury and lead in nine species from Anzali wetland and Hamoon wetland as indicators of natural and anthropogenic impacts on aquatic ecosystem. These wetlands are on the Ramsar list and are considered to be at risk. So far, such studies have not been conducted on the fish in these wetlands.
